# Phototheranostics of CD44-positive cell populations in triple negative breast cancer

**DOI:** 10.1038/srep27871

**Published:** 2016-06-15

**Authors:** Jiefu Jin, Balaji Krishnamachary, Yelena Mironchik, Hisataka Kobayashi, Zaver M. Bhujwalla

**Affiliations:** 1 Division of Cancer Imaging Research, The Russell H Morgan Department of Radiology and Radiological Science, The Johns Hopkins University School of Medicine, Baltimore, Maryland, USA; 2 Molecular Imaging Program, Center for Cancer Research, National Cancer Institute, US National Institutes of Health, Bethesda, Maryland, USA; 3 Sidney Kimmel Comprehensive Cancer Center, The Johns Hopkins University School of Medicine, Baltimore, Maryland, USA

## Abstract

Triple-negative breast cancer (TNBC) is one of the most lethal subtypes of breast cancer that has limited treatment options. Its high rates of recurrence and metastasis have been associated, in part, with a subpopulation of breast cancer stem-like cells that are resistant to conventional therapies. A compendium of markers such as CD44^high^/CD24^low^, and increased expression of the ABCG2 transporter and increased aldehyde dehydrogenase (ALDH1), have been associated with these cells. We developed a CD44-targeted monoclonal antibody photosensitizer conjugate for combined fluorescent detection and photoimmunotherapy (PIT) of CD44 expressing cells in TNBC. The CD44-targeted conjugate demonstrated acute cell killing of breast cancer cells with high CD44 expression. This cell death process was dependent upon CD44-specific cell membrane binding combined with near-infrared irradiation. The conjugate selectively accumulated in CD44-positive tumors and caused dramatic tumor shrinkage and efficient elimination of CD44-positive cell populations following irradiation. This novel phototheranostic strategy provides a promising opportunity for the destruction of CD44-positive populations that include cancer stem-like cells, in locally advanced primary and metastatic TNBC.

Breast cancer is the second most commonly diagnosed cancer and the second leading cause of death among women in the US^1^. Of the various breast cancer subtypes, triple-negative breast cancer (TNBC) is a highly aggressive and malignant form^2^. TNBC is defined as the subgroup of tumors that lacks expression of the estrogen receptor (ER) and progesterone receptor (PR), and lacks HER2 overexpression^3^. TNBC constitutes approximately 12 to 17% of all breast cancers and is characterized by poor prognosis and limited treatment options^3^,^4^. Since endocrine and HER2-targeted therapies are ineffective in TNBC, cytotoxic chemotherapy remains the mainstay of systemic treatment for TNBC patients^2^,^3^. However, despite an initial response to conventional chemotherapy that is frequently accompanied by collateral damage to normal tissues, these tumors relapse, display refractory drug-resistance, and metastasize earlier than other subtypes^2^. Several emerging targeted therapeutic agents, such as poly (ADP-ribose) polymerase inhibitors^5^,^6^, angiogenesis inhibitors^7^, and EGFR-targeted agents^8^ are being actively investigated in clinical trials in patients with TNBC, but there continues to be an unmet need for effective precision medicine of TNBC.

TNBC cells can survive chemotherapy and bypass the cellular apoptotic response to chemotherapy by undergoing alternative viable cellular fates, such as cellular senescence and cytoprotective autophagy^9^. The existence of a subpopulation of breast cancer stem cells (CSCs) that are resistant to conventional therapies may also contribute to the high rates of recurrence and metastasis of TNBC^10^. CSCs are defined as a population of tumor-initiating or propagating cells possessing the ability to self-renew and differentiate^11^, and are identified by a collection of cell surface makers such as CD44^high^/CD24^−/low^/Lin^− ^12,13 or CD44^+^/CD24^−^/EpCAM^+^ in breast cancer^10^. CD44^high^/CD24^−/low^ human breast CSCs are more abundant in TNBC patients than those with non-triple-negative tumors and their presence is associated with poor treatment outcome^14^. CD44 is a transmembrane glycoprotein receptor that plays a role in cell adhesion^15^. CD44 expression is up-regulated in hypoxic microenvironments^16^. CD44 is overexpressed in aggressive cancers^17^, making it an important target to eliminate aggressive breast cancer cell populations.

Therapeutic monoclonal antibodies (mAbs) have become an increasingly important category of targeted therapeutic agents in oncology^18^,^19^,^20^. However, high doses of mAbs are required to achieve satisfactory therapeutic outcomes. Thus, there are increasing reports of using low dose mAbs as carriers to deliver potent therapeutic agents, for example, phototoxic agents for targeted photodynamic therapy (PDT)^21^,^22^. Unfortunately, most commonly used PDT agents are hydrophobic, tend to aggregate in aqueous solutions after conjugation with mAbs, and emit in visible light with low tissue penetration^23^. Moreover, once exposed to light, PDT agents cause cell death by generating reactive oxygen species (ROS). PDT-induced cell death requires the internalization of PDT agents into organelles to achieve high phototoxic potency^24^. Human breast CSCs contain less ROS levels due to the up-regulation of the oxidative response genes in free radical scavenging systems, which leads to the resistance of breast CSCs to apoptotic death from ROS-dependent therapies such as PDT^25^. A novel form of PIT was recently developed by conjugating a photosensitizer, IR700, which is a near-infrared (NIR) phthalocyanine dye with excellent water-solubility and photo-stability, to mAbs targeting epidermal growth factor receptors (EGFR)^26^. The photoimmunoconjugate (PIC) demonstrated a profound ability for EGFR-specific cell killing and tumor shrinkage after NIR irradiation in preclinical models^26^,^27^,^28^,^29^,^30^,^31^. Distinct from conventional PDT, IR700-based PIT does not require intracellular delivery of the therapeutic agent, and exerts phototoxic effects only when adequate NIR irradiation and cell membrane binding are combined. Here we built upon this strategy to eliminate CD44 expressing cancer cells that include the CSC population, by using CD44 as a therapeutic target in a TNBC xenograft model. We performed cellular and *in vivo* studies to demonstrate and verify the specificity and efficacy of this novel CD44-specific PIT and investigated the underlying cell killing mechanism. As far as we know, this is the first demonstration of targeting CD44 cancer cell populations by PIT in TNBC. The NIR emission of IR700 has the added benefit of allowing noninvasive fluorescence detection to optimize the timing of NIR PIT for theranostic PIT.

## Results

### Characterization of CD44-IR700

**Figure 1:**
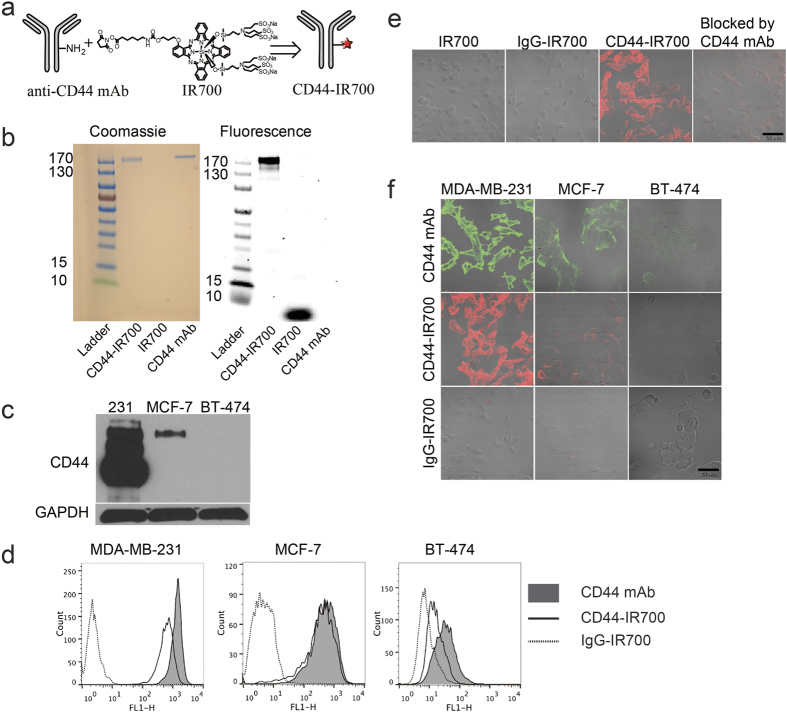
Characterization and CD44-specific cell binding of CD44-IR700. (**a**) Schematic illustration of CD44-IR700 synthesis. (**b**) Samples were run on SDS-PAGE gel and stained with Coomassie Blue, and checked with NIR fluorescence (λex = 633 nm, λem = 650~700 nm). (**c**) Immunoblot probing of CD44 expression in cells. (**d**) Flow cytometric analysis showing CD44-specfic binding of CD44-IR700. Cell lines were first incubated with CD44 mAb, CD44-IR700, or IgG-IR700 and then stained with Alexa 488 labeled anti-mouse IgG. (**e**) Confocal microscopy images of overlaid fluorescence and bright-field views of MDA-MB-231 cells following 1-h incubation with 0.2 μM of IR700, 10 μg/ml of IgG-IR700, 10 μg/ml of CD44-IR700, or 10 μg/ml of CD44-IR700 together with 40 μg/ml of CD44 mAb at 37 °C. (**f**) Confocal microscopy images of overlaid fluorescence and bright-field views of various cell lines after 1-h incubation with CD44 mAb, CD44-IR700, or IgG-IR700 at a concentration of 10 μg/ml at 3 °C. Scale bars indicate 50 μm.

The schematic in Fig. 1a depicts the preparation of CD44-IR700 through the attachment of NHS-activated IR700 to the free amine residues on CD44 mAb. After removing unbound IR700 moieties, we measured an average of three IR700 molecules conjugated to one CD44 mAb by UV spectroscopy. CD44-IR700 and control agents were loaded onto a gradient gel for SDS-PAGE. The images shown in Fig. 1b confirmed the high purity of CD44-IR700 that was free of unbound IR700 molecules. The strong association of IR700 with CD44 antibody in CD44-IR700, without any noticeable dissociation under SDS-PAGE conditions was also confirmed.

### CD44-specfic binding of CD44-IR700 *in vitro*

Five breast cancer cell lines, CD44-expressing triple negative MDA-MB-231, SUM149 and SUM159, ER positive MCF-7, and HER2 positive BT-474, were selected for *in vitro* studies. Representative immunoblots shown in Fig. 1c and Supplementary Fig. S1 demonstrate the relative CD44 expression level in the whole cell lysate of these five cell lines. High expression of CD44 in MDA-MB-231, SUM149 and SUM159 cells, low expression in MCF-7 cells, and undetectable expression in BT-474 cells was evident in the immunoblot. We used flow cytometry to characterize CD44 expression levels on the surface of these cells. Cells stained by CD44 mAb (black curves with filled area in Fig. 1d) had average intensities of 1401, 614, and 48 for MDA-MB-231, MCF-7, and BT-474 cells, respectively. CD44-IR700 (solid line) retained a slightly decreased binding affinity to the cell surface as unconjugated CD44 mAb, while IgG-IR700 (dotted line) was within the negative control range. We analyzed the CD44^+ ^/CD24^−^ subpopulation in five breast cancer cell lines by flow cytometry. The data in Supplementary Figs S2 and S3 demonstrate that the percentages of the CD44^+^/CD24^−/low^ subpopulation were 95.1%, 1.69%, 0%, 89.9%, and 92.1% for MDA-MB-231, MCF-7, BT-474, SUM149, SUM159 cells, respectively. We also performed confocal microscopy to illustrate CD44-specific binding of CD44-IR700 in cells. Representative images in Fig. 1e show IR700 fluorescence images of MDA-MB-231 cells following incubation with dye or conjugate at a concentration of 10 μg/ml or the equivalent concentration. IR700 or IgG-IR700-treated cells had no detectable IR700 fluorescence, while CD44-IR700-treated cells showed strong NIR fluorescence localized on the cell surface, which was largely blocked by co-incubation with a 4-fold higher concentration of CD44 mAb, implying that the binding of CD44-IR700 to the cell surface was CD44 receptor dependent. We performed confocal microscopy of all three cell lines with 1-h incubation of the following reagents: 10 μg/ml of CD44 mAb followed by Alexa Fluor 488-labeled secondary antibody; 10 μg/ml of IgG-IR700 or CD44-IR700 (Fig. 1f). As evident in the representative images in the panels, following CD44 mAb and CD44-IR700 incubation, the green fluorescence from CD44 mAb and NIR fluorescence from CD44-IR700 were highest in MDA-MB-231 cells followed by MCF-7 cells with the least in BT-474 cells, consistent with the flow cytometry results. The IgG-IR700 panel in Fig. 1f demonstrates negligible non-specific binding of the conjugates.

### CD44-IR700-mediated PIT selectively kills CD44 expressing triple negative MDA-MB-231, SUM149 and SUM159 breast cancer cells

**Figure 2:**
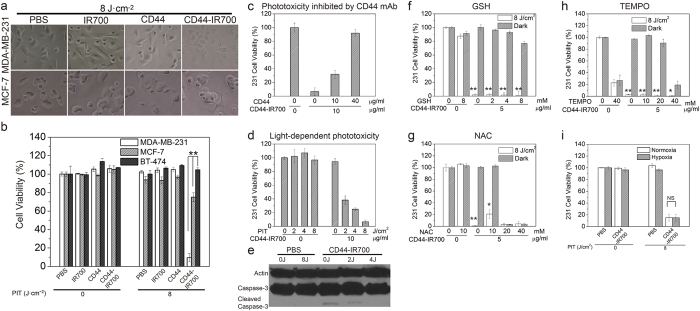
CD44-specific phototoxicity of CD44-IR700 and cell death mechanism. (**a**) Microscopic bright field images of MDA-MB-231 cells (upper) and MCF-7 cells (lower) after incubation with dye, antibody, or conjugate at 10 μg/ml or the equivalent amount for 1 h at 37 °C and following irradiation at 8 J/cm^2^. (**b**) Cell viability of various cell lines after incubation with dye, antibody, or conjugate with or without irradiation (**P < 0.01 for phototoxicity of CD44-IR700-irradiated MDA-MB-231 cells compared to other cells using Student’s *t* test). (**c**) CD44-IR700-mediated phototoxicity is inhibited by excess CD44 mAb in a dose-dependent manner. (**d**) CD44-IR700-mediated phototoxicity is dependent on the irradiation dose. (**e**) Western-blot to detect caspase-3 activation in CD44-IR700-induced cell death. (**f–h**) Cell viability of MDA-MB-231 cells in the presence of various ROS scavengers, GSH, NAC or TEMPO (*P < 0.05 and **P < 0.01 for viability of CD44-IR700-irradiated MDA-MB-231 cells compared to non-irradiated cells using Student’s *t* test). (**i**) Cell viability of MDA-MB-231 cells after incubation with PBS or 10 μg/ml of CD44-IR700 under hypoxia or normoxia. Values shown in (**b–d**) and (**f–h**) represent mean ± SD from triplicate experiments.

We examined bright field images of irradiated cells following incubation with 10 μg/ml of CD44-IR700, together with controls. As shown in Fig. 2a and Supplementary Fig. S4a, CD44-IR700 in combination with irradiation of 8 J/cm^2^ caused cellular swelling, membrane vesicle rupture and cell death in almost all the MDA-MB-231, SUM149 and SUM159 cells within 30-min after irradiation, while under the identical treatment, the majority of MCF-7 cells (approximately 80%) remained viable as evident from their intact morphology (Fig. 2a). We did not observe any morphological changes in either MDA-MB-231 or MCF-7 cells treated with control reagents, including PBS, IR700, or CD44 mAb, with or without light irradiation. We then measured the cellular toxicity of CD44-IR700 and controls in all five cell lines. As shown in Fig. 2b and Supplementary Fig. S4b, irradiation at 8 J/cm^2^ resulted in 91%, 62%, and 90% of cell death in CD44-IR700-treated MDA-MB-231, SUM149 and SUM159 cells, respectively, while 70% of MCF-7 cells and 99% of BT-474 cells were still alive. The control experiments showed that irradiation itself was harmless to cells and there was no cytotoxicity associated with IR700 or CD44 mAb with or without irradiation, or with CD44-IR700 in the absence of irradiation. The phototoxicity of CD44-IR700 towards MDA-MB-231, SUM149, SUM159 cells was inhibited by excess CD44 mAb in a dose-dependent manner (Fig. 2c, Supplementary Fig. S4c), and was dependent on the light irradiation dose (Fig. 2d, Supplementary Fig. S4d) and the concentration of CD44-IR700 in a dose-dependent manner (Supplementary Figs S4e and S5).

### Mechanism of cell death induced by CD44-IR700-mediated PIT

Apoptosis is a common cause of cell death with conventional PDT. We therefore evaluated caspase-3 activation in CD44-IR700-mediated PIT. As shown in Fig. 2e, cleaved caspase-3 was not observed in CD44-IR700-treated NIR-irradiated MDA-MB-231 cells, while a slight activation of caspase-3 was only observed in CD44-IR700-treated MDA-MB-231 cells without irradiation. Cell blebbing and rapid cell killing observed with CD44-IR700-mediated PIT, strongly suggests that PIT-induced cell death was mainly through necrosis and not apoptosis. Molecular oxygen is essential in conventional PDT to generate ROS for cell killing. We used ROS scavengers during incubation to remove ROS and tested their effects on CD44-IR700-mediated PIT. Three antioxidant reagents were chosen in our study. NAC (N-acetyl cysteine) is the most general broad spectrum antioxidant; glutathione (GHS) is required for many enzymatic antioxidant processes endogenous to the cell; 4-hydroxy TEMPO (TEMPOL), is a chemical scavenger of superoxide, the parent molecule of all ROS. All the ROS scavengers were added into the culture medium containing 5 μg/ml of CD44-IR700 at 1 hour prior to NIR exposure. The final concentrations of ROS scavengers were adjusted to different values, GSH: 0–8 mM, NAC: 0–40 mM, TEMPOL: 0–40 mM. As shown in Fig. 2f–h, only NAC (a broad spectrum antioxidant) at 10 mM partially inhibited CD44-IR700-induced cell death by approximately 20%, implying that molecular oxygen did not play a major role in photocytotoxicity from CD44-IR700-mediated PIT. We also examined the effect of hypoxia on the phototoxicity associated with CD44-IR700. As shown in Fig. 2i, under hypoxia, CD44-IR700 retained potent phototoxicity towards MDA-MB-231 cells, resulting in approximately 90% of cell death, comparable to that observed under normoxia.

### CD44-specfic tumor accumulation of CD44-IR700

**Figure 3:**
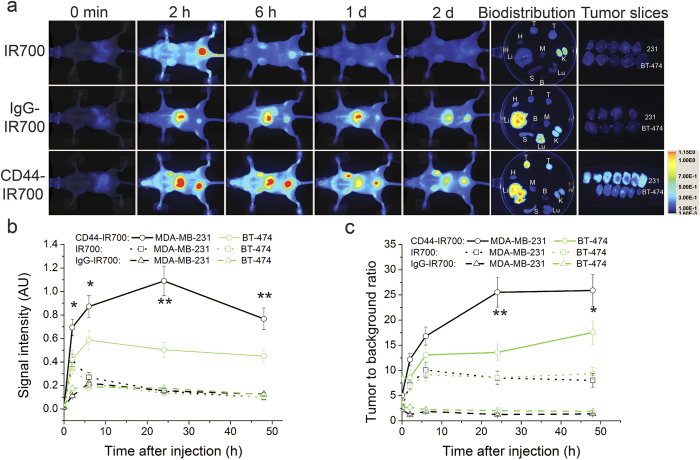
Preferential accumulation of CD44-IR700 in CD44-high tumors. (**a**) NIR fluorescence *in vivo* imaging of nude mice bearing bilateral tumors (upper, MDA-MB-231; lower, BT-474) over a 48-h period and fluorescence images of tumors, tumor slices and main organs at 48-h *p.i.* 50 μg of CD44-IR700 or IgG-IR700 or 1 nmol of IR700 injected *i.v.* In the petri-dish are MDA-MB-231 tumor (left), BT-474 tumor (right). Notations: T, tumor; H, heart; Li, Liver; S, spleen; Lu, lung; K, kidney; B, blood; M, muscle. (**b**) The IR700 fluorescence intensity and (**c**) the corresponding tumor to background ratio of MDA-MB-231 and BT-474 tumors over 48 hours after injection of compounds described in (**a**). Values represent mean ± SD (n = 4 per group, *P < 0.05, **P < 0.01 for MDA-MB-231 tumors from CD44-IR700-injected mice compared to the contralateral BT-474 tumors and MDA-MB-231 tumors from other control groups using Student’s *t* test).

To study the tumor-specific accumulation of CD44-IR700, we carried out NIR fluorescence imaging of nude mice bearing bilateral tumors (in the images MDA-MB-231 is the upper tumor and BT474 is the lower tumor) before and after intravenous (*i.v.*) injection of 50 μg of CD44-IR700 or IgG-IR700 or 1 nmol of IR700 (n = 4 per group). As shown in representative images in Fig. 3a, CD44-IR700 accumulated extensively in the MDA-MB-231 tumor compared to the BT-474 tumor. NIR fluorescence in the MDA-MB-231 tumor increased steadily after injection, reached a maximum at 1 day post-injection (*p.i.*), and decreased slightly at day 2. Strong NIR fluorescence signal from CD44-IR700 was detected in the liver, indicating hepatobiliary clearance of CD44-IR700, which is typical of antibody clearance. Accumulation of IR700 or IgG-IR700 in either MDA-MB-231 or BT-474 tumors was not observed over this time. The bladder or liver retention of NIR signals in IR700- or IgG-IR700-injected mice suggests renal and hepatobiliary clearance, respectively, of these two compounds. To more precisely illustrate the differential uptake of the compounds in the tumors, tumors were excised at 2 days *p.i.* and sectioned into 1-mm tumor slices. Tumor slices were scanned to obtain and quantify the fluorescence images. Also shown in Fig. 3a are representative *ex vivo* fluorescence images obtained at 2 days *p.i.* demonstrating the distribution of the compounds in major organs and tumors. The biodistribution results confirm the preferential accumulation of CD44-IR700 in MDA-MB-231 tumors, and confirm the clearance patterns of the compounds observed *in vivo*. The resulting data confirm the specificity of CD44-IR700 to MDA-MB-231 tumors and demonstrate almost no uptake of IR700 or IgG-IR700 in either MDA-MB-231 or BT-474 tumors. To further examine the MDA-MB-231 tumor specificity of CD44-IR700, we performed a semi-quantitative analysis of *in vivo* fluorescence images to determine the dynamic changes in signal intensity of all the compounds in the tumors (Fig. 3b) and as tumor to background ratio (TBR, Fig. 3c) over a two-day period. In CD44-IR700-injected mice, MDA-MB-231 tumors exhibited a two-fold increase in both fluorescence signal intensity and TBR at 1 day *p.i.* when compared to the contralateral BT-474 tumors. In IR700- and IgG-IR700-injected mice, the fluorescence signals from all the tumors were within the background level, and TBR values in IgG-IR700 or IR700-injected mice were much lower than values from CD44-IR700 injected mice.

### CD44-IR700-mediated PIT selectively inhibits the growth of MDA-MB-231 tumors

**Figure 4:**
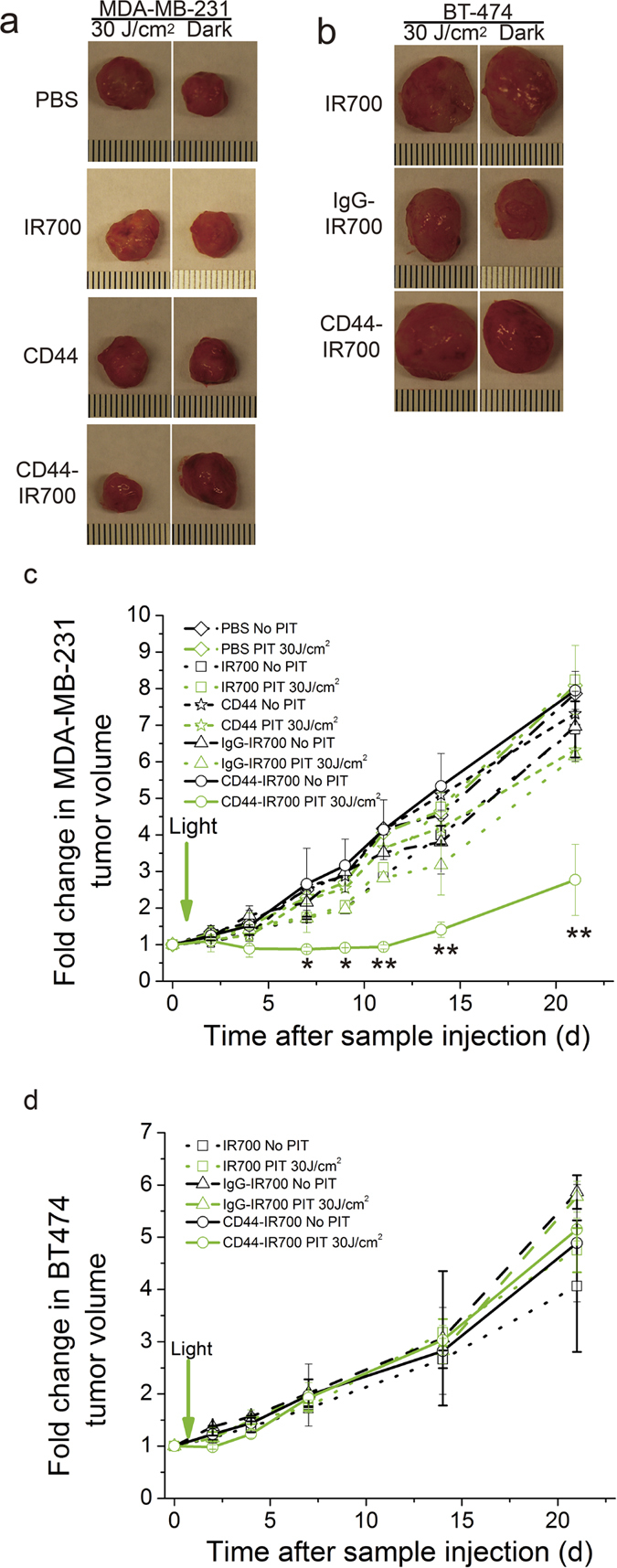
CD44-IR700-mediated PIT *in vivo*. Representative photographs of excised tumors from nude mice bearing (**a**) bilateral MDA-MB-231 tumors or (**b**) bilateral BT-474 tumors at the end of 3-week treatment. A single *i.v.* injection of 100 μg of CD44-IR700 or IgG-IR700 or CD44 mAb or 2 nmol of IR700 was delivered. One tumor (left) was exposed to NIR irradiation at 30 J/cm^2^ with a wavelength of 680 nm at 24-h *p.i.*, and the contralateral one (right) was covered by aluminum foil. The corresponding tumor growth curves of (**c**) MDA-MB-231 tumors and (**d**) BT-474 tumors over 3 weeks *p.i.* Values represent mean ± SD (n = 4 or 5 per group, *P < 0.05, **P < 0.01 for the irradiated MDA-MB-231 tumors from CD44-IR700-injected mice compared to other control groups using Student’s *t* test).

We next examined the efficacy and selectivity of CD44-IR700-mediated PIT *in vivo*. Mice bearing bilateral MDA-MB-231 tumors were randomly assigned to five groups (n = 5 per group) receiving *i.v.* injection of 100 μg or the equivalent amount of the agents: (i) PBS; (ii) IR700; (iii) IgG-IR700; (iv) CD44 mAb; (v) CD44-IR700. The left side tumor was exposed to 30 J/cm^2^ of NIR light at 24-h *p.i.,* and the right one was covered with aluminum foil. We monitored tumor growth over a 3-week period. In CD44-IR700-injected mice, NIR irradiation significantly inhibited MDA-MB-231 tumor growth, while the right side tumor without irradiation grew normally, as evident from the significant size difference of excised tumors (Fig. 4a) and distinctly different tumor growth curves (Fig. 4c). In control agent-injected mice, MDA-MB-231 tumors on both sides grew rapidly whether or not irradiation was given, as seen from the comparable sizes of excised tumors and similar tumor growth patterns (Fig. 4a,c).

**Figure 5:**
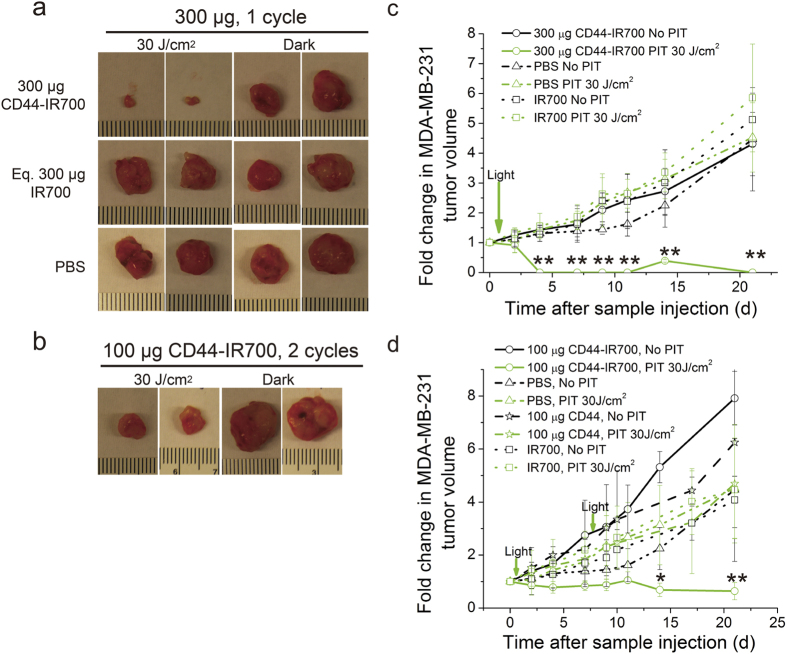
*In vivo* PIT with different dosing strategies. (**a**) Representative photographs of excised tumors (two from each group) from nude mice bearing bilateral MDA-MB-231 tumors at the end of 3-week treatment. A single *i.v.* injection of 300 μg of immunoconjugates/controls was dosed. (**b**) Representative photographs of excised tumors (two from each group) from nude mice bearing bilateral MDA-MB-231 tumors at the end of 3-week treatment. Two *i.v.* injections (at one week interval) of 100 μg of CD44-IR700 were given. One tumor (left) was exposed to NIR irradiation at 30 J/cm^2^ with a wavelength of 680 nm at 24-h p.i., and the contralateral one (right) was covered by aluminum foil. The tumor growth curves of 300 μg injection (**c**) and two i.v. injections (**d**) over 3 weeks *p.i.* Values represent means ± SD (n = 4 per group, *P < 0.05, **P < 0.01 for the irradiated MDA-MB-231 tumors from CD44-IR700-injected mice compared to other control groups using Student’s *t* test).

We performed the same study on the bilateral BT-474 tumor model. We did not observe growth delay in CD44-IR700-treated irradiated BT-474 tumors that demonstrated a similar growth profile as CD44-IR700-treated unirradiated tumors or control agent-treated tumors (Fig. 4b,d). We then changed the CD44-IR700 dosing strategy and examined the effect on tumor growth by using the bilateral MDA-MB-231 tumor model. When the dose of CD44-IR700 was increased to 300 μg for a one-time injection, the irradiated MDA-MB-231 tumor shrank dramatically, and only a minimal amount of tumor tissue remained at the end of treatment, while the unirradiated tumor on the right was not affected, and showed a tumor growth pattern similar to the control agent-treated tumors (Fig. 5a,c). When CD44-IR700 was given in two injections of 100 μg each with one week interval in between, we again observed significantly enhanced tumor growth inhibition comparable to the single injection protocol (Fig. 5b,d). Better therapeutic efficacy of CD44-IR700 in MDA-MB-231 tumors was achieved by increasing the dosage or introducing more treatment cycles.

### Immunohistochemistry studies

**Figure 6:**
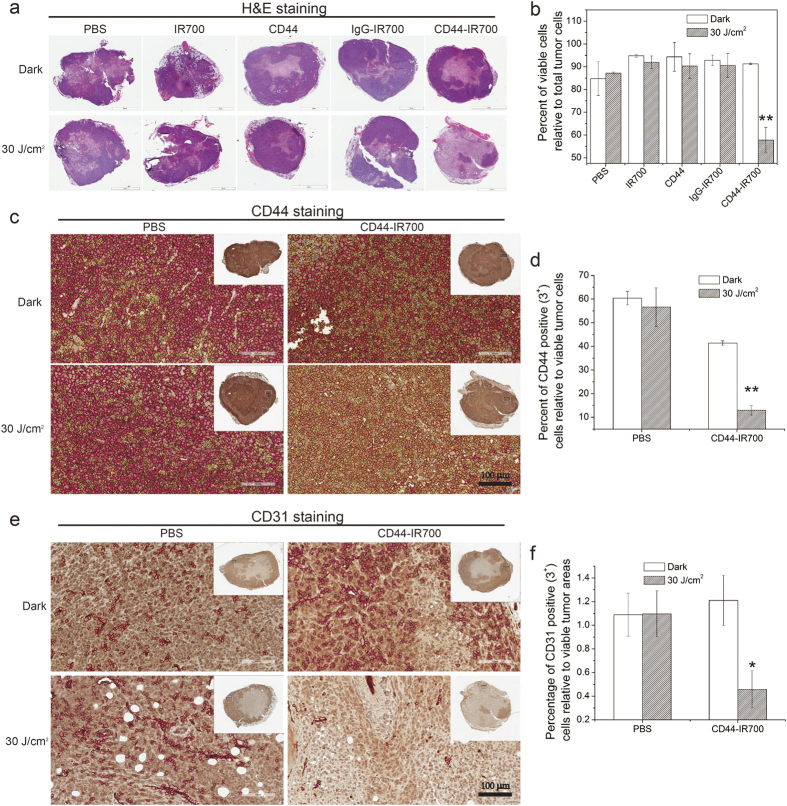
H&E and IHC characterization of tumors for necrosis, CD44 and CD31. (**a**) Representative images of tumor sections after H&E staining. Nude mice bearing bilateral MDA-MB-231 tumors received a single injection of 100 μg of immunoconjugates/controls with or without irradiation at 24-h *p.i.*, and tumors were excised at 4 days *p.i.* (n = 3 per group). (**b**) Quantitative analysis of viable cells in tumor sections from H&E stained images. Representative images of tumor sections after CD44 (**c**) or CD31 (**e**) immunostaining and the corresponding quantitative analysis of the percentage of CD44 positive (3^+^) cells relative to the viable tumor cells (**d**) and the percentage of CD31 positive (3^+^) vasculature cells relative to the viable tumor area (**f**). Values represent means ± SD (n = 3 per group, *P < 0.05, **P < 0.01 for the tumor slices from CD44-IR700-injected NIR-irradiated mice compared to non-irradiated control groups using Student’s *t* test). In (**c,e**), red, orange and yellow circles mark 3^+^, 2^+^ and 1^+^ positive cells, respectively. Scale bars indicate 100 μm.

To further examine the tumor response to CD44-IR700-mediated PIT, histological analysis was performed on MDA-MB-231 tumors resected from CD44-IR700-treated mice and other control agent-treated mice at 4 day *p.i.* In the CD44-IR700-treated unirradiated tumors, the H&E stained images of tumor sections clearly demonstrated viable cells with some central necrosis as shown in the representative images in Fig. 6a. In contrast, CD44-IR700-mediated PIT resulted in profound cell death in the tumor tissue, with only a small focus of MDA-MB-231 cells remaining viable after irradiation as seen in the representative H&E section from a CD44-IR700-treated irradiated tumor. Quantification of viable cells is shown in Fig. 6b and demonstrates that ~90% of total tumor cells were viable as determined by H&E staining in CD44-IR700-treated unirradiated tumor slices, while this percentage declined to 57% in irradiated slices. The percentage of viable cells in other control agent-treated tumor slices was approximately 90%, whether or not tumors were irradiated. To investigate the effect of CD44-IR700-mediated PIT on CD44 expression and tumor vasculature, we examined the expression levels of CD44 and CD31 in viable regions of all tumor slices as identified from H&E stained images. The data shown in Fig. 6c–f clearly demonstrate the significant decrease of CD44 and CD31 expression in CD44-IR700-treated tumor slices after NIR irradiation, while the CD44 and CD31 expression levels in unirradiated tumor slices were comparable to PBS-treated tumors with or without irradiation.

## Discussion

Achieving precision cancer medicine where only cancer cells are eliminated with minimum collateral damage to normal cells continues to remain an important unmet need. The plasticity and resilience of cancer cells is exemplified in tumor subpopulations with stem-like characteristics that significantly contribute to resistance to treatment and recurrence^10^,^11^. By targeting CD44, these stem-like subpopulations in breast cancers can be eliminated by a treatment that is not susceptible to drug resistance and induces catastrophic cell death only when CD44-IR700 bound to the CD44 receptor is exposed to NIR irradiation. Neither component on its own induces cell damage, which is important since CD44 receptors are expressed in epithelial cells^32^. Skin toxicity of bivatuzumab mertansine, a toxin-conjugated mAb targeting CD44v6, was observed in Phase I clinical trails in patients with head and neck squamous cell carcinoma^33^,^34^. However, radioimmunotherapy studies with the anti-CD44v6 conjugates Re-cmAb U36 and Re-bivatuzumab achieved promising antitumor effects without skin toxicity^35^,^36^,^37^, implying that the supertoxicity of mertansine may have caused the skin toxicity^34^. Since NIR-PIT is effective only in regions irradiated by NIR light, based on fluorescent imaging, the light dose and area can be optimized to maximize therapeutic effects and minimize possible side effect to the skin or precursors of T-cells in the thymus that may express CD44. This phototheranostic strategy provides an opportunity to selectively eliminate aggressive CD44 populations in primary and metastatic TNBC, and for those TNBCs that uniformly express CD44, the opportunity to eliminate the entire population of cancer cells in the tumor.

IR700 is a NIR-emissive phthalocyanine dye with excellent water solubility and photo-stability. It exhibits a strong absorption at 689 nm, within the “phototherapeutic window” (650–900 nm), where the NIR light is able to penetrate deeper, to achieve better depths for tumor imaging and photo-treatment. Although the orthotopic breast cancer xenografts we used were relatively superficial, IR700-based PIT has already been applied to peritoneal disseminated tumors^38^,^39^ and lung cancer metastasis^40^,^41^. With endoscopic delivery of NIR irradiation or intraoperative applications, this novel NIR-PIT strategy can treat deep-seated tumors or the tumor bed during surgery. Furthermore, the fluorescence of IR700 can be used to monitor body distribution and tumor accumulation to select the optimum timing for photo-irradiation.

A dose of 50 μg CD44-IR700 was used for *in vivo* imaging since higher TBR was observed with injection of 50 μg compared to 100 μg CD44-IR700 in MDA-MB-231 tumors. These results are consistent with a previous study^26^ that demonstrated a higher TBR with 50 μg of Pan-IR700 compared to 300 μg of Pan-IR700. The difference of TBR between MDA-MB-231 and BT-474 tumors was approximately two-fold higher, although CD44 expression levels in the immunoblot were several-fold higher in MDA-MB-231 cells compared to BT-474 cells. This difference may reflect differences in the total amount of standard form CD44 protein detected in cell lysates compared to CD44 protein on the cell surface where binding of CD44-IR700 occurs. Another explanation for the difference between CD44 expression levels in cells and targeted *in vivo* imaging data may be due to differences in enhanced permeability and retention (EPR) in MDA-MB-231 and BT-474 tumors.

The specificity of CD44-IR700 PIT to eliminate CD44 positive cells was evident from the dramatic reduction of tumor growth observed in MDA-MB-231 tumors but not in BT474 tumors following CD-IR700 PIT. Approximately 85 ± 5% of MDA-MB-231 cells express CD44 and almost none of the BT-474 cells are CD44-postive^42^. The hepatobiliary clearance pathway of CD44-IR700, resulted in a relatively high retention of CD44-IR700 in the liver but since the liver was not exposed to NIR irradiation, the presence of CD-IR700 alone was not phototoxic.

Conventional PDT requires the internalization of photosensitizers into organelles such as mitochondria or lysosome, to achieve better phototoxic effects. Intracellular delivery is not required for CD44-IR700-mediated PIT. Additionally, hypoxic tumor regions may have reduced ROS species that can impact PDT^43^. Hypoxia has also been found to upregulate CD44 expression^16^. CD44-IR700-mediated PIT retained potent phototoxicity under hypoxia, and may serve as an attractive treatment to destroy hypoxic cancer cells that are typically resistant to radiation and chemotherapy and display a more aggressive phenotype^44^.

We found that CD44-IR700-mediated PIT treatment eliminated the bulk of CD44-positive cancer cells in the tumor. CD44-IR700-mediated PIT also decreased CD31 expression levels indicating significant destruction of vasculature that may have also contributed to the profound decrease of tumor growth and catastrophic cell death in the CD44 positive treated tumors. CD44 was recently identified as a novel marker for vasculogenic tumor cells; the reduction of CD44 expression suppressed vascular formation and targeting CD44 attenuated tumor angiogenesis^17^.

Patient-derived tumor xenograft (PDX) animal models, established by transplanting human cancer tissue into a mouse host, are the preferred preclinical models in anti-cancer drug discovery^45^. Mouse PDX models are more accurate for mimicking clinical trails than our currently used xenograft model established by inoculating human cancer cells but they are more challenging to handle. Our phototheranostic agent CD44-IR700 is expected to inhibit tumor growth and eliminate CD44-positive cell population by phototherapy in TNBC PDX models as long as CD44-overexpression is validated on the surface of patient-derived tumor cells. Future studies with patient derived xenograft (PDX) models will provide additional data to support the translational applications of these studies. CD44-IR700-mediated PIT may provide the opportunity of eliminating residual TNBC cells intraoperatively. In locally advanced breast cancer, CD44-IR700 PIT may provide an approach of shrinking tumors that do not respond to chemotherapy prior to surgery in the neoadjuvant setting.

In summary, we developed a CD44-targeted mAb photosensitizer complex (APC) for combined fluorescent detection and PIT treatment of CD44-positive populations of TNBC. The acute and severe phototoxic effects of CD44-IR700, take place as long as two requirements-adequate NIR light and cell membrane binding, are met. This novel cell killing pathway together with the crucial role of CD44 in breast cancer make CD44-IR700-mediated PIT a promising treatment option for effective and precise treatment of CD44 expressing TNBCs. In addition to the CSC marker of breast cancer^12^,^13^, CD44 has also been identified as a cancer stem cell marker in pancreatic^46^, prostate^47^, ovarian^48^, and colon cancer^49^, making it a significantly important molecule for targeting. The CD44 targeting approach developed here will be applicable not only to TNBC but to a wide range of cancers where CD44 is a significant target.

## Methods

### Materials

Water soluble phthalocyanine dye, IRDye 700DX NHS ester (IR700) was obtained from Li-Cor Bioscience (LI-COR Biosciences, Lincoln, Nebraska). Anti-CD44 monoclonal IgG1 antibody with clone MEM-263 (CD44 mAb) and IgG from mouse serum (IgG) were obtained from Sigma (St. Louis, MO). *N*-acetyl-L-cysteine (NAC), L-glutathione reduced (GSH), and 4-hydroxy-TEMPO (TEMPOL) were purchased from Sigma. All other reagents were of reagent grade.

### Synthesis and characterization of IR700-conjugated anti-CD44 monoclonal antibody

In a typical antibody-photosensitizer conjugate synthesis, 1 mg of CD44 mAb was first dispersed in 1 ml of 1X PBS and then added with 100 μl of K_2_HPO_4_ buffer (pH = 9.0) and 159.2 μg of IR700 (81.6 nmol, 1 mM in DMSO). The mixture was kept at 4 °C for overnight, and then placed in ultra-centrifugal filter units (Millipore Amicon Ultra-0.5, 10 kDa, Billerica, MA) to remove the unbound IR700 molecules. The IR700-conjugated anti-CD44 monoclonal antibody obtained was abbreviated as CD44-IR700. Similarly, we obtained mouse IgG conjugated with IR700 as a non-target control, which was abbreviated as IgG-IR700. The concentration of antibody and dye/protein ratio was determined spectroscopically by measuring the absorbance of the conjugate at 280 nm and 689 nm. The extinction coefficients were 210,000 M^−1^cm^−1^ for CD44 mAb at 280 nm, and 165,000 M^−1^cm^−1^ for IR700 at 689 nm. The correction factor of IR700 at 280 nm was set to be 0.095. The purity of CD44-IR700 was checked with sodium dodecyl sulfate polyacrylamide gel electrophoresis (SDS-PAGE) performed on a 4–20% gradient gel (Mini-PROTEAN TGX precast gels, BIO-RAD, Hercules, CA). Fluorescence from the gel was obtained from a Typhoon gel scanner (GE Healthcare Bio-Sciences, Piscataway, NJ), and the protein bands on the gel were stained with GelCode^®^ blue stain reagent (Thermo Fisher Scientific, Grand Islands, NY).

### Cell culture

Five wild-type human breast cancer cells, MDA-MB-231, MCF-7, BT-474, SUM149, and SUM159 cell lines were purchased from American Type Culture Collection (ATCC, Manassas, VA). MDA-MB-231, MCF-7, and BT-474 cells were cultured in 10% fetal bovine serum (FBS, Sigma) supplemented RPMI 1640 (Sigma), MEM (Mediatech Inc, Manassas, VA) and ATCC 46-X (ATCC) media, respectively. SUM149 and SUM159 cells were cultured in DMEM/Ham’s F-12 medium 50/50 Mix (Mediatech Inc) with 5% FBS, 5 μg/ml insulin and 1 μg/ml hydrocortisone. Cells were maintained at 37 °C in a humidified atmosphere containing 5% CO_2_.

### Fluorescence microscopy

Cells were seeded onto an 8-well Lab-Tek II chamber slide (Nalge Nunc, Rochester, NY) at a density of 10,000 cells/well one day prior to imaging. Once cells reached 70~80% confluence, cells were incubated with the corresponding culture media containing CD44-IR700 or IgG-IR700 at a concentration of 10 μg/ml for 1 h at 37 °C. To validate the dependence of CD44-IR700 binding on CD44, MDA-MB-231 cells were incubated with CD44-IR700 containing medium supplemented with excess of CD44 mAb at 40 μg/ml. After replenishing the wells with fresh culture medium, cells were imaged with a laser scanning confocal microscope (Zeiss LSM 510-Meta, Carl Zeiss Microscopy GmbH, Jena). The red laser at 633 nm was used for excitation, and the receiving PMT channel was set at 680~700 nm. All the images were obtained under identical microscope settings.

### Flow cytometry

Cells were detached using 1X non-enzymatic cell dissociation solution (Sigma) and washed and suspended in FACS buffer (1X PBS, 1%BSA, 0.1% NaN_3_). To examine the expression levels of CD44 and the binding affinity and specificity of CD44-IR700, 1 × 10^6^ of live cells were first incubated with 2 μg/ml of CD44 mAb, CD44-IR700, or IgG-IR700 at 4 °C for 1 h, and secondarily stained by 2 μg/ml of Alexa 488 labeled goat anti-mouse secondary antibody (1:1000, Life Technologies, Grand Island, NY) at 4 °C for 30 min. To analyze the CD44^+^/CD24^−/low^ subpopulation in various breast cancer cells, 1 × 10^6^ of live cells were incubated with FITC-conjugated mouse anti-human CD24 (clone ML5, BD Biosciences, San Jose, CA) and APC-conjugated mouse anti-human CD44 (clone G44-26) or their respective isotype controls (BD Biosciences) at 4 °C for 30 min. Fluorescence of the cells was acquired on FACS Calibur (BD Bioscience) and five thousand events of cells were analyzed and processed using FlowJo software (FLOWJO, LLC, Ashland, OR).

### *In vitro* NIR phototherapy and phototoxicity assay

Ten thousand cells were seeded onto 96-well plates and incubated for 24 h. Medium was replaced with the corresponding fresh medium containing the following reagents: PBS, 0.2 μM of IR700, 10 μg/ml CD44 mAb or CD44-IR700. The cells were further incubated for 1 h at 37 °C. After washing with PBS, phenol red-free medium was added. A light emitting diode (Marubeni, Santa Clara, CA) provided NIR irradiation to the cells at the peak wavelength of 680 nm and irradiation at 8 J/cm^2^. Immediately after NIR irradiation, 10 μl of CCK-8 reagent (Dojindo, Rockville, MD) was added to each well of the plate and further incubated with cells at 37 °C for 3 h. For controls, cells exposed to the same incubation conditions were kept in the dark by wrapping plates with aluminum foil. The absorbance of the cell medium was measured at 450 nm on a Victor3^TM^ multilabel plate reader (Perkin Elmer, Waltham, MA). The power density of the LED was measured by an optical power meter (PM 100, Thorlabs). The cytotoxicity data were expressed as mean ± standard derivation (SD) from triplicate trials.

To study the dose-dependence of CD44-IR700-mediated phototoxicity in three TNBC cell lines, the incubation conditions for MDA-MB-231, SUM149 and SUM159 cells were set by varying the CD44-IR700 concentration but maintaining NIR exposure, or by changing the NIR exposure while maintaining CD44-IR700 concentration.

To study the CD44-dependence of CD44-IR700-mediated phototoxicity in three TNBC cell lines, CD44 mAb at different concentrations were added together with CD44-IR700 to co-incubate MDA-MB-231 cells, SUM149 and SUM159 cells.

To study the role of ROS in CD44-IR700-mediated phototoxicity, a selection of ROS scavengers were added into the culture medium containing 5 μg/ml of CD44-IR700 at 1 hour prior to NIR exposure. The final concentrations of ROS scavengers were adjusted to different values, GSH: 0–8 mM, NAC: 0–40 mM, TEMPOL: 0–40 mM. MDA-MB-231 cells without NIR exposure or in the absence of CD44-IR700 were used as controls. To study the effect of hypoxia on CD44-IR700-mediated phototoxicity, hypoxia was induced by placing the plates in a modular incubator chamber (Billups-Rothenberg, Del Mar, CA), flushed at 2 p.s.i. for 3 minutes with a gas mixture of 0.2% O_2_, 5% CO_2_, and N_2_ for the balance. MDA-MB-231 cells were incubated with 10 μg/ml of CD44-IR700 or PBS and maintained under hypoxia for 1 h at 37 °C followed by NIR irradiation also under hypoxia, after which cytotoxicity was assayed using CCK-8.

### Animal model and tumor implantation

All the animal care and *in vivo* procedures were conducted in accordance with the regulations dictated by the Institutional Animal Care and Use Committee of The Johns Hopkins University. The Institutional Animal Care and Use Committee approved the animal protocol. Six to eight-week-old female athymic Balb/c (nu/nu) mice were purchased from Charles River (NCI, Fredrick, MD). Tumor models were established by inoculating 2 × 10^6^ MDA-MB-231 or BT-474 cells in 0.05 ml of Hanks balanced salt solution into the upper-right mammary fat pad. To determine the tumor volume, the length (longitudinal diameter), width (transverse diameter), and depth of the tumor was measured with an external caliper, and the ellipsoidal tumor volume was calculated as length × width × depth × 0.52.

### *In vivo* and *ex vivo* fluorescence imaging

Fluorescence imaging of tumor-bearing mice was performed on a Li-Cor Pearl^®^ Impulse imager (LI-COR Biosciences). Nude mice with bilateral MDA-MB-231 and BT-474 tumors, inoculated in the mammary fat pad were selected for imaging when tumors reached 100 mm^3^. 50 μg of CD44-IR700 or IgG-IR700 or 1 nmol of IR700 was injected *i.v.*, and the fluorescence images of IR700 in the mice were obtained over a 2-day period (n = 4 per group). All the fluorescence images were acquired under identical experimental conditions. Regions of interest (ROIs) were drawn on the tumors in the fluorescence images and analyzed by Pearl Impulse software (LI-COR Biosciences) to obtain fluorescence intensity. At 48 h post injection, mice were euthanized, and main organs and tumors were isolated and placed in a 10-cm petri-dish for imaging. To quantitatively determine the tumor uptake by fluorescence, fresh tumors were subsequently cut into 1-mm slices with a slicer and imaged with an Odyssey^®^ scanner (LI-COR Biosciences).

### *In vivo* photoimmunotherapy

Mice bearing bilateral MDA-MB-231 tumors were randomly assigned to five groups (n = 5 per group) receiving *i.v.* injection of different agents of 100 μg or the equivalent amount: (i) PBS; (ii) IR700; (iii) IgG-IR700; (iv) CD44 mAb; (v) CD44-IR700, once the tumor volume reached 50 mm^3^. To compare the different dosing strategies, MDA-MB-231 tumor bearing mice were randomized into three groups (n = 4 per group) that received the following treatments: (i) 100 μg of CD44-IR700, one cycle; (ii) 300 μg of CD44-IR700, one cycle; (iii) 100 μg of CD44-IR700, two cycles. To study the target specificity of the CD44-IR700-mediated PIT *in vivo*, nude mice were inoculated with bilateral BT-474 tumors and randomized into three groups (n = 4 per group) for *i.v.* injection of 100 μg or the equivalent amount of following agents: (i) IR700; (ii) IgG-IR700; (iii) CD44-IR700. The left side tumor (mouse placed prone) was exposed to NIR irradiation at 30 J/cm^2^ (provided by a LED from Marubeni) at a wavelength of 680 nm at 24-h p.i., and the right one was covered by aluminum foil. All the mice were monitored over a 3 week-period by checking fluorescence with a Li-Cor Pearl^®^ Impulse imager, taking photos with a digital camera, and measuring tumor diameters with a caliper. After three weeks of monitoring, mice were euthanized, and the tumors were excised.

### Immunohistochemistry (IHC) study

For IHC studies, mice bearing bilateral MDA-MB-231 tumors received the same treatments (five groups, n = 3 per group) as those in the *in vivo* PIT group with the only exception that tumors were resected at 4 days *p.i.* The excised tumors were fixed in 10% neutral buffered formalin and paraffin-embedded. 10-μm thick tumor sections were cut and stained with hematoxylin and eosin (H&E), or immunostained with anti-mouse CD31 antibody (Rat monoclonal antibody, DIA-310, clone SZ31Dianova, 1:30 dilution, Dianova, Hamburg, Germany) and anti-human CD44 antibody (mouse monoclonal,Ab-4, Clone156-3C11, 1:70 dilution, Thermo Scientific, Fremont, CA) according to standard IHC protocols. All the stained IHC slides were scanned and analyzed by Aperio ImageScope software with well-selected algorithms (Leica Biosystems Inc., Buffalo Groove, IL).

### Statistical analysis

Data were expressed as mean ± SD from at least three samples or animals. Statistical analysis was performed with one-sided student t-test (Microsoft Excel), assuming unequal variance. Values of *P* ≤ 0.05 were considered significant, unless otherwise stated.

## Additional Information

**How to cite this article**: Jin, J. *et al*. Phototheranostics of CD44-positive cell populations in triple negative breast cancer. *Sci. Rep.* **6**, 27871; doi: 10.1038/srep27871 (2016).

## Supplementary Material

Supplementary Information
