# Supplementary material for: Phototheranostics of CD44-positive cell populations in triple negative breast cancer
Source: Sci Rep. 2016 Jun 15;6:27871. doi: 10.1038/srep27871 (PMC4908597; doi:10.1038/srep27871)
Supplement: Supplementary Information [file srep27871-s1.doc]

**Supplementary Information for Phototheranostics of CD44-positive cell populations in triple negative breast cancer**

Jiefu Jin^1^, Balaji Krishnamachary^1^, Yelena Mironchik^1^, Hisataka Kobayashi^3^, Zaver M. Bhujwalla^1,2*^

*^1^Division of Cancer Imaging Research, The Russell H Morgan Department of Radiology and Radiological Science, ^2^Sidney Kimmel Comprehensive Cancer Center, The Johns Hopkins University School of Medicine, Baltimore, Maryland, USA; ^3^Molecular Imaging Program, Center for Cancer Research, National Cancer Institute, US National Institutes of Health, Bethesda, Maryland, USA*

* Correspondence should be addressed to: [zaver@mri.jhu.edu](mailto:zaver@mri.jhu.edu)


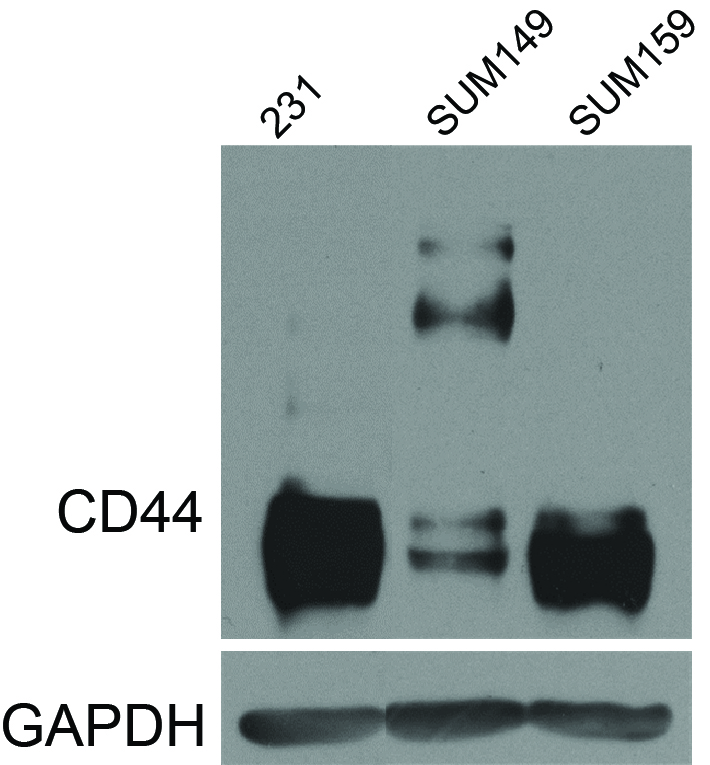


**Figure S1.** Western blot analysis of CD44 expression in MDA-MB-231, SUM149, and SUM159 cells.


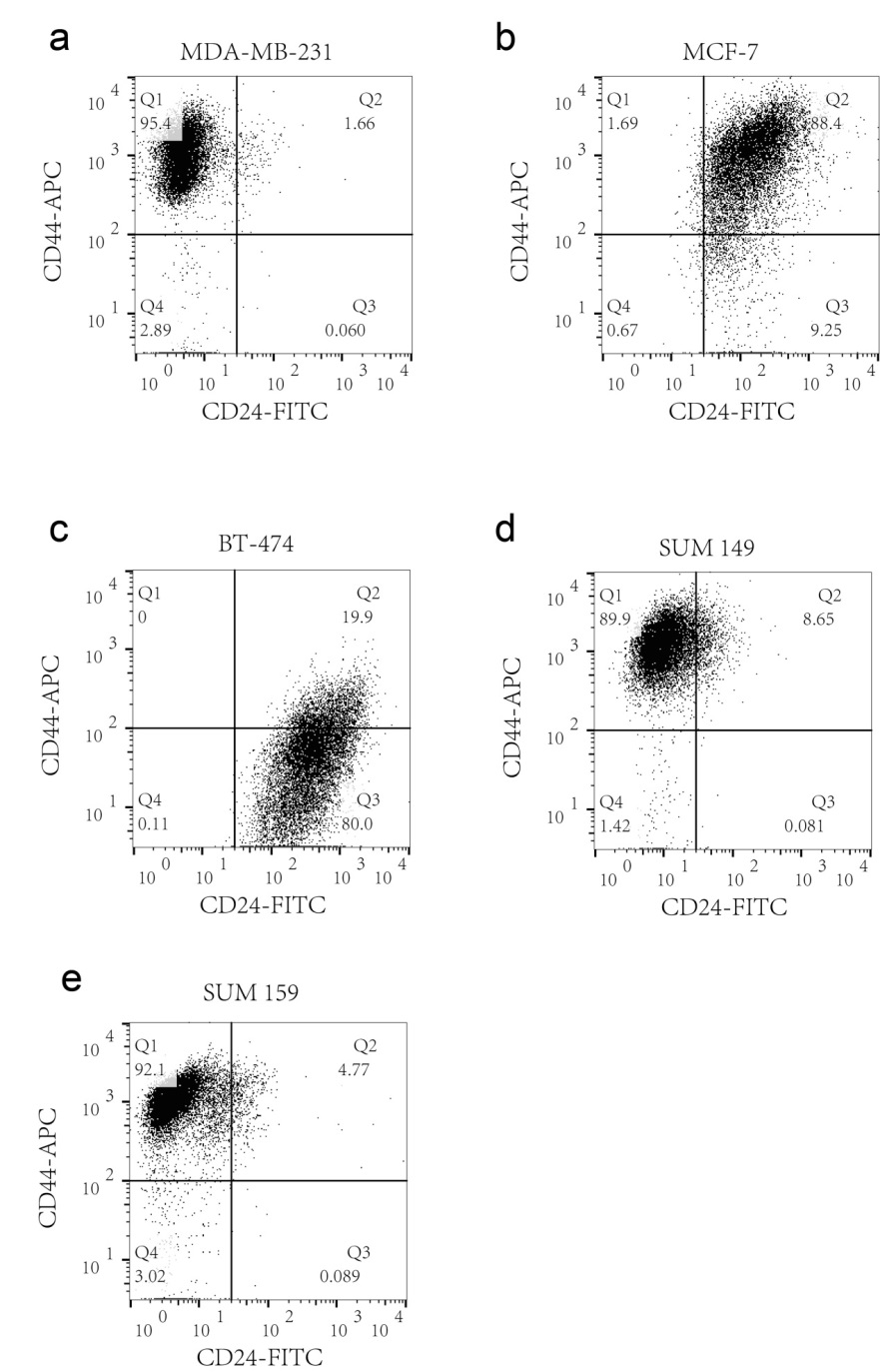


**Figure S2. Identification of CD44^+^/CD24^-^ subpopulation in breast cancer cell lines by flow cytometry.**

Five breast cancer cell lines, (a) MDA-MB-231, (b) MCF-7, (c) BT-474, (d) SUM149, and (e) SUM159 were stained with FITC-conjugated mouse anti-human CD24 and APC-conjugated mouse anti-human CD44 monoclonal antibodies. Cells in Q1 correspond to CD44^+^/CD24^-/low^ cells.

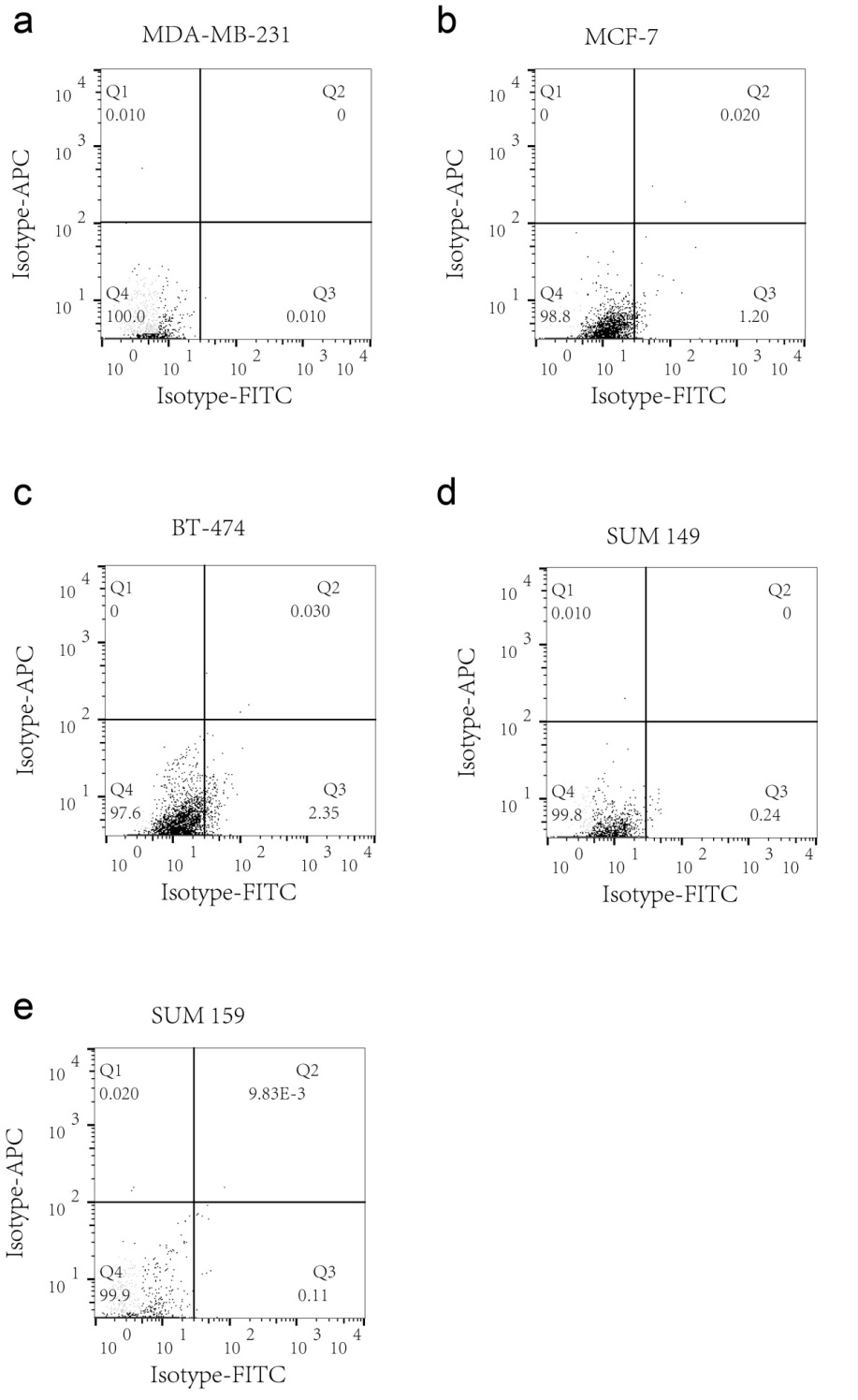

**Figure S3.** Flow cytometric analysis of five breast cancer cell lines after staining with isotype controls.


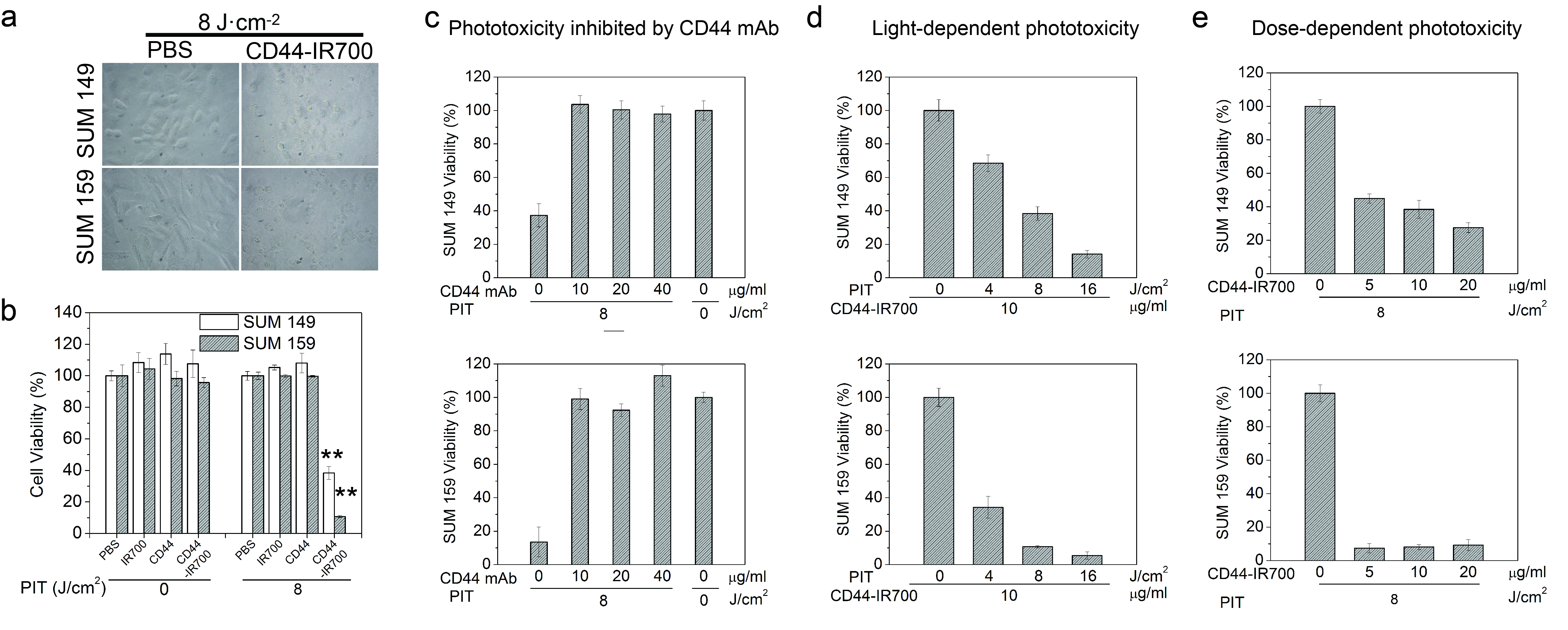


**Figure S4. CD44-specific phototoxicity of CD44-IR700 on SUM149 and SUM159 cells.**

(a) Microscopic bright field images of SUM149 cells (upper) and SUM159 cells (lower) after incubation with PBS or 10 μg/ml of CD44-IR700 for 1 h at 37 °C and following irradiation at 8 J/cm^2^. (b) Cell viability of SUM 149 and SUM159 cells after incubation with dye, antibody or conjugate with or without irradiation (**P<0.01 for phototoxicity of CD44-IR700-irradiated SUM cells compared to non-irradiated cells using Student’s *t* test). (c) CD44-IR700-mediated phototoxicity in SUM cells inhibited by excess CD44 mAb. (d) CD44-IR700-mediated phototoxicity in SUM cells is light dose-dependent. (e) CD44-IR700-mediated phototoxicity in SUM cells is conjugate dose-dependent. Values shown in (b-e) represent mean ± SD from quadruple experiments.


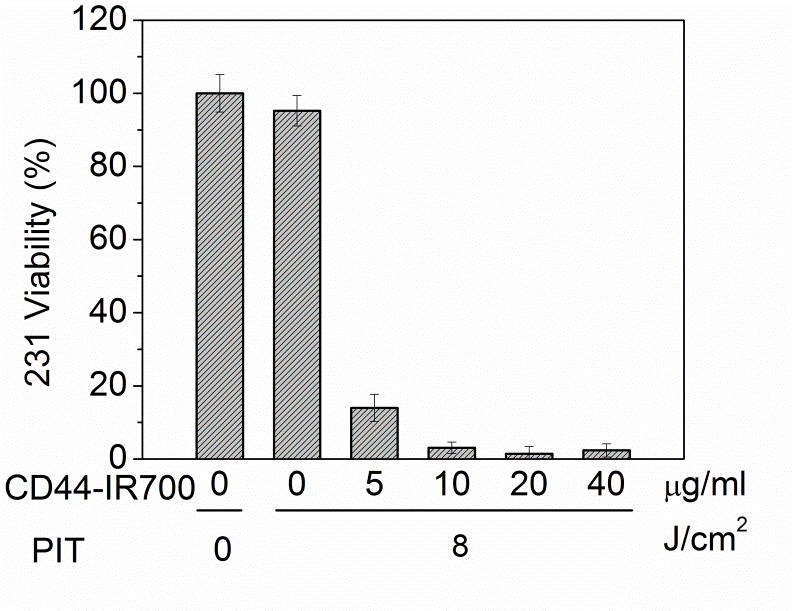


**Figure S5.** CD44-IR700-mediated phototoxicity in MDA-MB-231 cells is dependent on the dose of the antibody-dye conjugate.
